# Strigolactones affect phosphorus acquisition strategies in tomato plants

**DOI:** 10.1111/pce.14169

**Published:** 2021-08-25

**Authors:** Veronica Santoro, Michela Schiavon, Ivan Visentin, Christian Constán‐Aguilar, Francesca Cardinale, Luisella Celi

**Affiliations:** ^1^ DISAFA Department University of Turin Grugliasco Italy; ^2^ Department of Plant Physiology, Faculty of Sciences University of Granada Granada Spain

**Keywords:** organic acid anions, phosphatase, phosphate transporters, phosphorus, phytase, rhizosphere acidification, root exudation kinetics, strigolactones, tomato, uptake

## Abstract

Strigolactones (SLs) are plant hormones that modulate morphological, physiological and biochemical changes as part of the acclimation strategies to phosphorus (P) deficiency, but an in‐depth description of their effects on tomato P‐acquisition strategies under P shortage is missing. Therefore, in this study, we investigate how SLs impact on root exudation and P uptake, in qualitative and quantitative terms over time, in wild‐type and SL‐depleted tomato plants grown with or without P. Under P shortage, SL‐depleted plants were unable to efficiently activate most mechanisms associated with the P starvation response (PSR), except for the up‐regulation of P transporters and increased activity of P‐solubilizing enzymes. The reduced SL biosynthesis had negative effects also under normal P provision, because plants over‐activated high‐affinity transporters and enzymatic activities (phytase, acidic phosphatase) to sustain elevated P uptake, at great carbon and nitrogen costs. A shift in the onset of PSR was also highlighted in these plants. We conclude that SLs are master kinetic regulators of the PSR in tomato and that their defective synthesis might lead both to suboptimal nutritional outcomes under P depletion and an unbalanced control of P uptake when P is available.

## Abstract 2

In this study, we aimed to evaluate the role of strigolactones (SLs) in modulating a broad spectrum of phosphorus (P) acquisition and utilization strategies in tomato (*Solanum lycopersicum* L.) plants. Indeed, most information about SLs and P nutrition is available for the model plant *Arabidopsis thaliana*. Our work clearly shows that SLs silencing causes suboptimal nutritional outcomes under P depletion and unbalanced control of P uptake under P availability in tomato plants, and that SLs kinetically regulate P starvation responses in this crop.

## 1 INTRODUCTION

Phosphorus (P) is one of the essential elements that plants require to develop and function. Phosphate rock reserves are limited, and much of the P supply in agricultural soils is not bioavailable after application of P‐based fertilizers, due to fixation reactions that sequester P making it one of the most immobile, inaccessible and unavailable among all nutrient elements (Holford, 1997; Niu et al., 2013; Richardson, Barea, McNeill, & Prigent‐Combaret, 2009). Phosphorus is absorbed and assimilated by plants as inorganic phosphate (Pi), which occurs at fairly low concentrations in the soil solution, typically in the 1–10 μM range (Bieleski, 1973; Hinsinger, 2001). This value is well below the tens of μM required for optimal plant growth (Aziz et al., 2014; Péret, Clément, Nussaume, & Desnos, 2011). Therefore, even though many soils are high in P, only a small portion of the P pool is promptly available for plant uptake (Richardson et al., 2009; Schachtman, Reid, & Ayling, 1998).

Plants have evolved an array of adaptive strategies to optimize P usage and increase P acquisition under P deficiency that are collectively known as P‐starvation response (PSR) (de Souza Campos et al., 2019) and encompass growth, development and metabolic adjustments (Péret et al., 2011). Processes aimed to conserve P in plants involve, among others, decreased growth rate and increased growth per unit of P uptake (also referred to as P‐utilization efficiency, PUtE), P internal recycling and adoption of metabolic pathways that do not involve P‐requiring reactions (Vance, Uhde‐Stone, & Allan, 2003). Instead, mechanisms targeting P‐acquisition efficiency (PAE) imply greater allocation of photosynthates to the root apparatus to promote root growth and exudation, modifications of root system architecture, and regulation of P transporters (Lynch, 2007; Marschner et al., 1995; Richardson et al., 2011; Schachtman et al., 1998; Vance et al., 2003). Changes in root growth and root system architecture, in particular, are among the best‐documented plant responses to P starvation, but show some degrees of species specificity. Maize (*Zea mays* L.) and *Arabidopsis thaliana*, for example, adopt the strategy of increasing lateral root number when P‐depleted (Dixon, Simonne, Obreza, & Liu, 2020), whereas tomato (*Solanum lycopersicum* L.) plants show such a response under high P concentration (2 mM) (Jiang, Chen, Xu, Zhu, & Yao, 2015). On the other hand, tomato plants can respond to P deficiency by increasing the root surface area, and decreasing the total root weight and average root diameter (Garcia & Ascencio, 1992; Santoro et al., 2020).

Root exudation of low molecular weight organic acids and protons is a general strategy to enhance P mobilization and acquisition from the soil (Marschner, 1995). Organic acids released by roots can mobilize both inorganic and organic P species by complexing metal cations that bind P, while displacing P from the soil matrix by ligand exchange (Lambers, Shane, Cramer, Pearse, & Veneklaas, 2006). Secretion of phosphatases or phytases may also promote P release from organic moieties (Czarnecki, Yang, Weston, Tuskan, & Chen, 2013). In addition, plants can enhance the expression of high‐affinity P transporters (PHTs) that function at the plasma membrane of root cells to improve P foraging (Lambers et al., 2006; Rouached, Arpat, & Poirier, 2010; Shen et al., 2011; Siao, Coskun, Baluška, Kronzucker, & Xu, 2020).

Among the systemic adaptive plant responses that regulate P homeostasis and the efficient use of stored P, the reallocation of P from old to young and/or actively growing tissues (Schachtman et al., 1998; Shen et al., 2011) and P recycling from P‐containing metabolites, like phospholipids (Ticconi & Abel, 2004; Vance et al., 2003), are widely observed processes. Plant P homeostasis is also regulated by the conserved microRNA 399 (miR399), which acts as a long‐distance signal highly responsive to low P conditions (Czarnecki et al., 2013), and is thought to be a crucial node of the PSR (Pant, Buhtz, Kehr, & Scheible, 2008). The molecular target of miR399 is the transcript of *PHOSPHATE2* (*PHO2*), encoding an ubiquitin‐conjugating enzyme that mediates the degradation of Phosphate Starvation‐Induced (PSI) gene products, like PHO1 and PHT family member proteins (Liu et al., 2012).

Strigolactones (SLs) are emerging as phytohormones with a role in plant adjustments to P levels. Several studies support the existence of a tight interplay of the SL signalling node with the biosynthetic and regulatory networks of other phytohormones (reviewed in Yang, Lian, & Wang, 2019). Strigolactones are a group of carotenoid‐derived terpenoid lactones, initially identified as germination stimulants of parasitic plants (Cook, Whichard, Turner, Wall, & Egley, 1966), then as hyphal‐branching factors for arbuscular mycorrhizal fungi (AMF) (Akiyama, Matsuzaki, & Hayashi, 2005), and more recently as plant hormones (Gomez‐Roldan et al., 2008; Umehara et al., 2008). Studies carried out with *Arabidopsis*, pea and rice mutants flawed in SL production or signalling revealed that these compounds act as long‐distance branching factors suppressing axillary bud outgrowth (Gomez‐Roldan et al., 2008; Umehara et al., 2008). Underground, their effects on root growth and development are intertwined with that of the sibling pathway initiated by KARRIKIN INSENSITIVE2 (KAI2) and are seen on primary root growth, lateral and adventitious root formation and root‐hair length and density (Czarnecki et al., 2013; Koltai, 2013; Villaécija‐Aguilar et al., 2019). Under P‐deficiency conditions, SLs appear to mediate multiple levels of morphological, physiological and biochemical changes as part of the acclimation strategies to optimize plant growth (Brewer, Koltai, & Beveridge, 2013; Czarnecki et al., 2013; Santoro et al., 2020). High production and exudation of SLs under P stress conditions are proposed to repress shoot branching, increase lateral root formation and root hair density in rice and *Arabidopsis*, as well as promote AMF symbiosis in host plants (Brewer et al., 2013). Recently, it has been observed that P‐starved, SL‐depleted tomato plants show a dramatic decrease in root hair elongation and lateral root number and length, and clear alterations of root tip anatomy and morphology with extensive cell and tissue disorganization (Santoro et al., 2020). This suggests that a flawed SL metabolism affects tomato root plasticity under P starvation.

Tomato has become an ideal model species for SL studies, thanks to the availability of SL biosynthesis mutants and its agronomical importance all over the world. However, even though some aspects of SL‐mediated regulation of tomato responses to P stress have been addressed already (Gamir et al., 2020; Koltai et al., 2010; Santoro et al., 2020; Vogel et al., 2010), an in‐depth description of their effects on the plant P‐acquisition strategies under P shortage is still missing. Recently, Gamir et al. (2020) have indicated SLs as early modulators of tomato plant responses to low P availability, as the expression of P‐related key regulators (e.g., miR399 and PHO2) was induced after 1 hr pulse application of the synthetic SL analogue 2′‐*epi*‐GR24, highlighting the kinetic involvement of SLs in the regulation of P starvation signalling at early stages of deficiency (Gamir et al., 2020).

Our study is based on the hypothesis that P acquisition and allocation, as well as exudates production, may be affected by SLs in qualitative and quantitative terms over time. Therefore, we investigated the influence of SLs on P acquisition in wild‐type and SL‐depleted tomato plants, either grown with or without P, in terms of biomass production, P content and allocation, expression of PSI genes coding key PSR markers, such as PHT and PSR regulators (miR399 and *SlPHO2*). In addition, we contrasted the short‐ and long‐term kinetics of P uptake along with the kinetics of exudate release by measuring proton extrusion, dissolved organic carbon (DOC) and organic acid anions in the two genotypes. Finally, we assessed the effect of exogenous SLs on rhizosphere acidification, P‐acquisition and ‐utilization by wild‐type plants.

## 2 MATERIALS AND METHODS

### 2.1 Plant material and growth conditions

In this study, the tomato (*Solanum lycopersicum* L.) *SlCCD7‐*silenced line 6936 and its wild‐type genotype M82 were a kind gift by Dr. H. J. Klee (University of Florida); in the former, the production of the major SLs is reduced by about 80–90% with respect to the latter (Vogel et al., 2010). Seeds were surface sterilized in 70% (v/v) ethanol for 2 min, then in 3% NaClO for 20 min, washed five times for 10 min with sterile water, and then germinated on wet Whatman filter paper in Petri dishes (10 cm diameter) at 25°C and in darkness for 5 days. Germinated seeds were transferred to plastic pots filled with silica sand and allowed to grow for 45 days in a growth chamber with a 16/8 hr light/dark cycle, air temperature of 25°C and relative humidity ≥70%, with a light intensity of 100 μmol m^−2^ s^−1^. The silica sand was washed with 10% H_2_SO_4_ prior to use, to reach a background P concentration below the detection limit (0.1 mg P kg^−1^ sand). The nutrient solution, Hoagland modified, was renewed every day (1 mM MgSO_4_, 1 mM Ca(NO_3_)_2_, 250 μM KNO_3_, 80 μM KH_2_PO_4_, 20 μM FeNaEDTA, 9 μM H_3_BO_3_, 1.8 μM MnCl_2_, 0.2 μM ZnSO_4_, 0.2 μM Co(NO_3_)_2_, 0.2 μM NiSO_4_, 0.2 μM CuSO_4_, pH adjusted to 6.0).

### 2.2 Hydroponic growth and collection of root exudates and plant samples

After 45 days of growth in sand, wild‐type and SL‐depleted tomato plants were gently removed from the pots, and roots thoroughly washed with deionized water to remove any sand and P residue. Plants were then individually transferred into 250 ml flasks, each containing 200 ml of the nutrient solution aerated by an air pump. The flasks were covered with aluminium foils to prevent light from interfering with root growth and reduce photodegradation of labile organic compounds. Plants were kept with the full nutrient solution for 2 days to acclimatize and, before the application of the P treatments, roots were gently rinsed with deionized water several times. Half the plants were then kept in a P‐free (0 μM KH_2_PO_4_, named –P) nutrient solution, while the remaining plants were supplied with the complete nutrient solution containing 80 μM KH_2_PO_4_, hereafter called +P. KCl replaced KH_2_PO_4_ in the –P solution to provide plants with the same amount of K. Four biological replicates of one plant each were set up for each genotype and treatment. For 13 days, the growth solution was entirely collected at different time points and renewed. The collected solution was filtered by 0.22 μm nylon membrane filters, and stored at −20°C for further analyses. On day 13, plants were moved to 250 ml flasks containing deionized water in order to stimulate further exudation. Root exudates were collected after 1, 4, 8 and 24 hr in deionized water; then, shoots and roots were harvested separately, and the fresh weight biomass (FW) was recorded. Furthermore, the number of internodes was counted, and the weight of senescent leaves collected along the experiment was determined. Root subsamples were rapidly frozen with liquid N_2_ and stored at −80°C for enzymatic and gene expression analyses, while the remaining roots and shoots were dried at +40°C and total dry weight biomass (DW) was determined for each organ and plant. Dry root and shoot samples were ground separately, passed through a 0.5 mm mesh sieve, and used for elemental analyses.

### 2.3 Plant elemental analysis

Total carbon (C) and nitrogen (N) contents were determined by dry combustion (UNICUBE, Elementar Analysensysteme GmbH, Langensenbold, Germany). The P concentration in root and shoot tissues was determined colorimetrically on dry plant material (50 mg) after sulfuric‐perchloric digestion using the malachite green method (Ohno & Zibilske, 1991). Phosphorus‐acquisition efficiency was calculated as the ratio of P accumulated in tissues to exogenously supplied P during both plant growth in sand and the hydroponic experiment, while P‐utilization efficiency was calculated as the ratio of dry biomass to P content in plant tissues (Neto, Favarin, Hammond, Tezotto, & Couto, 2016). Root‐to‐shoot P translocation was indirectly estimated calculating the ratio of P content in the shoot divided by P content in the root.

### 2.4 Enzyme activity in root tissues

Enzymatic activity was assayed as in Hayes, Richardson, and Simpson (1999). Root material was ground in 15 mM 2‐(*N*‐morpholino)ethanesulfonic acid (MES) buffer (pH 5.5) containing 0.5 mM CaCl_2_·H_2_O and 1 mM EDTA. The extract was centrifuged at 13,800*g* × 15 min at 4°C and the supernatant subjected to gel filtration at 4°C on Sephadex G‐25 columns. To assay total acid phosphatase activity, the enzyme extract was incubated at 26°C in 15 mM MES buffer (pH 5.5) with 1 mM EDTA, 5 mM cysteine and 10 mM *p*‐nitrophenyl phosphate (*p*NPP). The assays were conducted for 30 min and the reactions stopped by addition of 0.25 M NaOH. The concentration of *p*‐nitrophenol (*p*NP) was determined by measuring the absorbance at 412 nm against standard solutions. Phytase activity was measured on the same root extracts and under the same conditions as described earlier, except that *p*NPP was replaced with 2 mM potassium *myo*‐inositol hexaphosphate (*myo*InsP6). Assays were conducted for 60 min and the reactions stopped by addition of ice‐cold 10% trichloroacetic acid (TCA). Solutions were subsequently centrifuged to remove precipitated material and phosphate concentration determined by the malachite green method (Ohno & Zibilske, 1991).

### 2.5 Gene transcript and microRNA quantification

To determine the levels of *SlPHO2* and *PHT* gene transcripts, and of mature miR399, RNA was extracted from individual root samples of wild‐type and SL‐depleted plants grown in hydroponics with either 80 μM KH_2_PO_4_ or no KH_2_PO_4_ as described earlier. Total RNA was extracted by using Spectrum™ Plant Total RNA Kit (Sigma‐Aldrich), and treated with DNase I (ThermoScientific) at 37°C for 30 min to remove residual genomic DNA. First‐strand cDNA was synthesized from 500 ng of purified total RNA using the High‐Capacity cDNA Reverse‐Transcription Kit (Applied Biosystems, Monza, Italy) according to the manufacturer's instructions. A modified protocol with a stem‐loop primer (Pagliarani et al., 2017) was followed for targeted miR399 cDNA synthesis. The stem‐loop primer and specific primer pairs are reported in Table S1. For transcript quantification of target genes, the quantitative reverse‐transcriptase PCR (qRT‐PCR) reactions were carried out in a StepOnePlus system (Applied Biosystems) using the SYBR Green (Applied Biosystems) method. Transcript concentrations were normalized on *SlEF‐1α* or *SlsnU6* transcripts as endogenous controls. Four independent biological replicates were analysed for each treatment, and each qRT‐PCR reaction was run in technical triplicates. Transcript amounts were quantified through the 2^−ΔΔCt^ method. The qRT‐PCR efficiency was calculated by using five‐fold serial dilutions of cDNA for each primer pair in separate amplification reactions. The means of threshold cycles were plotted versus the dilution factors in a logarithmic graph and the data fitted in a straight line. All the assays showed a correlation coefficient of 1 (± 0.1) and a slope between −3.6 and −3.3.

### 2.6 Phosphorus concentration in the nutrient solution and exudates analysis

The concentration of P in the nutrient solution was determined as described earlier, right before each sampling of root exudates, in order to evaluate the amount of P absorbed by plants.

Root exudates were then analysed for proton, DOC and organic acid anions content. The concentration of protons in the nutrient and water solutions was monitored using a pH‐sensitive electrode (inoLab pH 7110, WTW GmbH, Weilheim, Germany) and was expressed as the cumulative amount of H^+^ released at each sampling time. For the visualization of the acidification halo around root seedlings in separate experiments, half‐strength MS medium (Murashige & Skoog, 1962) at 0.625 mM KH_2_PO_4_, with or without the synthetic SL analogue *rac‐*GR24 (from StrigoLab, Turin, Italy, hereafter GR24; 5 μM in 0.01% acetone), was used for seedling growth after pre‐germination in sterile water for 3 days. Twelve‐day‐old wild‐type seedlings were then transferred on the same medium in which they had been germinated but without GR24 and amended with bromocresol purple (0.004% w/v, final pH: 7) (Planes et al., 2015). After 24 hr of further incubation in the same growth chamber, the plates were photographed, and the colour shift of the medium was compared with the corresponding pH scale for the bromocresol purple.

Dissolved organic C was determined using Pt‐catalysed, high‐temperature combustion (850°C) followed by infrared detection of CO_2_ (VarioTOC, Elementar, Hanau, Germany), after removing inorganic C by acidifying to pH 2 and purging with CO_2_‐free synthetic air. Data were expressed as mg C g^−1^ plant DW to evaluate the exudates in terms of C investment by the plant.

For organic acid anions determination, aliquots of root exudates (20 ml) were lyophilized and redissolved in etanhol/H_2_O (1/1 v/v), then analysed by Dionex DX‐500 Ion Chromatography system (Sunnyvale, CA) equipped with a dimensional‐exclusion column (Ion PAC ICE‐AS6) and an electrochemical detector (Dionex ED40), and data expressed as mg C g^−1^ plant as for DOC. Organic acid anions in the samples were identified by comparison with the retention times of pure standards including oxalic, tartaric, malic, lactic, acetic, maleic, citric, succinic and fumaric acid anions.

Protons, DOC and organic acid anions data at different time intervals were fitted with a proper mathematical equation (as reported in Table 3) in order to obtain the exudation kinetic parameters.

### 2.7 Effects of exogenous SLs on P acquisition efficiency in a greenhouse trial

To complement the study of SLs effect on P use efficiency, a parallel greenhouse experiment was set up, in which seeds of wild‐type plants were germinated as above and transplanted into 24‐cm‐diameter plastic pots (10 L) filled with peat and vermiculite (50:50 by volume) in the greenhouse. For each of the two trials performed, four plants were sprayed with GR24 (5 μM in 0.01% acetone in water) until drip‐off. In parallel, four other control plants were mock‐treated with the same solvent. The plants were treated thrice, at the growth stages of seedlings, adult vegetative and budding, after 9, 54 and 59 days from transplanting, respectively. In each group (GR24‐ or mock‐treated), the plants were watered twice a week with a half‐strength Hoagland solution containing 125 μM KH_2_PO_4_. Plants were harvested after 75 days of growth and FW, DW and total P content were quantified separately for roots and shoots as detailed earlier.

### 2.8 Statistics

For all determinations, the analysis of variance (one‐way ANOVA) was performed using the SPSS software version 26.0 (SPSS, Chicago, IL), and was followed by pair‐wise post‐hoc analyses (Student–Newman–Keuls test) to determine which means differed significantly at *p* < .05 (± SE, as indicated in figure legends). In addition, to evaluate the effect of plant genotype, P treatment and the combination of the two on the measured parameters, we used a linear mixed‐effect ANOVA model performed with the statistical programming language R (R Core Team, 2020). Figures were created using SigmaPlot ver. 12.5 software (Systat, San Jose, CA).

## 3 RESULTS

### 3.1 Tomato morphological plasticity in dependence of P supply

Strigolactone‐depleted plants displayed a marked increase in leaf nodes when compared with the wild‐type (Table 1, Figure S1). Although no significant differences were detected among treatments, both wild‐type and SL‐depleted plants showed the highest biomass values (FW) when grown with P supplement. Conversely, a non‐significant trend towards higher root DW values was observed in both genotypes in the absence of P, as expected, resulting in elevated root to shoot (R/S) ratios (Table 1). Since SL‐depleted plants grown under +P were inclined to produce less root biomass in favour of greater resource allocation to the shoot, they also displayed the lowest R/S ratio and a clear difference with the values under –P conditions. Wild‐type plants instead shared similar R/S ratios under either P growth conditions. Thus, the R/S ratios were significantly affected by the P treatment (*p* < .001) rather than the plant genotype.

**TABLE 1:** Effect of P treatments on fresh (FW) and dry biomass (DW), root/shoot ratio (R/S), number of nodes and senescent leaves of wild‐type (WT) and SL‐depleted (SL–) tomato plants after 13 days of hydroponic culture with (+P, 80 μM) or without (–P, 0 μM) Pi, followed by 24 hr in deionized water

	Plant FW (g)	Root DW (g)	Shoot DW (g)	R/S	Number of nodes	Senescent leaves DW (g)
WT +P	12.9 ± 1.1	0.22 ± 0.04	1.07 ± 0.11^ab^	0.20 ± 0.02^bc^	6.25 ± 0.25^b^	0.63 ± 0.16^ab^
WT –P	12.4 ± 0.9	0.27 ± 0.07	1.03 ± 0.24^ab^	0.27 ± 0.03^ab^	7.25 ± 0.25^b^	0.70 ± 0.19^a^
SL– +P	15.6 ± 1.1	0.20 ± 0.05	1.39 ± 0.30^a^	0.15 ± 0.01^c^	11 ± 0.91^a^	0.11 ± 0.00^bc^
SL– –P	10.9 ± 1.5	0.26 ± 0.09	0.91 ± 0.22^b^	0.28 ± 0.03^a^	10.75 ± 0.62^a^	0.02 ± 0.00^c^

*Note*: Each value represents the mean of four replicates (± SE). Different letters indicate significant differences between treatments (*p* < .05).

No significant variation in shoot biomass was observed in wild‐type plants depending on exogenous P supply, and only a slight, non‐significant increase in leaf senescence was observed (Table 1). Strigolactone‐depleted plants had lower relative amounts of biomass allocated to senescent leaves, a trend that was even more obvious under –P conditions (Table 1).

### 3.2 Phosphorus, C and N content and P‐use efficiency

**FIGURE 1:**
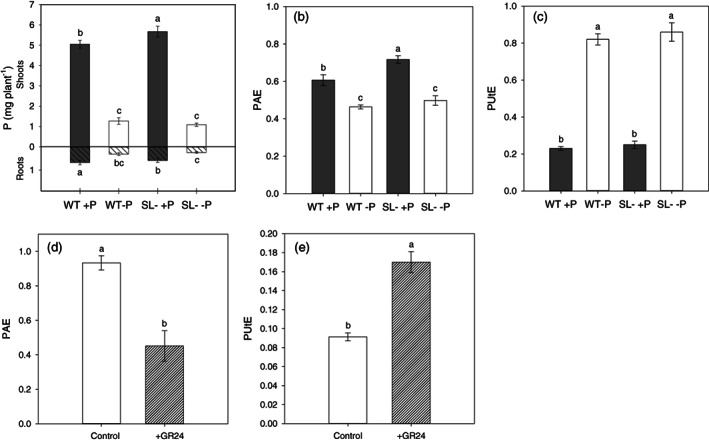
(a) Roots and shoots P content, (b) P‐acquisition efficiency (PAE) and (c) P‐utilization efficiency (PUtE) of wild‐type (WT) and SL‐depleted (SL–) tomato plants after 13 days of hydroponic culture with (+P, 80 μM) or without (–P, 0 μM) Pi, followed by 24 hr in deionized water. (d) PAE and (e) PUtE values in wild‐type plants grown in the greenhouse at 125 μM Pi and treated, or not, with 5 μM GR24. Each value represents the mean of four replicates (± SE). Different letters above bars indicate significant differences between treatments (*p* < .05)

Phosphorus sufficient plants accumulated about five times more P in their shoot than their relative –P genotypes (Figure 1a). The same trend was determined in roots, although differences in this case were less pronounced. Wild‐type and SL‐depleted plants showed a similar root‐to‐shoot P translocation factor (Figure S2). Strigolactone‐depleted plants contained more P in their shoot, consistent with the higher shoot biomass (Figure 1a). Conversely, wild‐type plants under +P retained more P in their roots, to which slightly but not significantly more biomass was allocated (Table 1). Under P shortage, the two genotypes had comparable amounts of P both in their shoots and roots (Figure 1a).

PAE was higher in +P than in –P tomato plants (Figure 1b). Maximal values were computed for SL‐depleted plants under +P, whereas no differences were found between plant genotypes grown under P deficiency. This parameter was strongly influenced by the P treatment (*p* < .001), the plant genotype (*p* < .01) and the combination of both factors (*p* < .05). PUtE (Figure 1c) was instead dependent only on the P treatment (*p* < .001) and its trend was opposite to that of PAE, as values were higher under –P than under +P conditions.

The results of the comparison between wild‐type and SL‐depleted plants are also complemented by the results obtained under SL supplementation, delivered as GR24 on the leaves of potted wild‐type plants in greenhouse conditions. Under these circumstances, PAE was significantly decreased (Figure 1d) and PUtE increased by GR24 treatment (Figure 1e), with shoot values being the main drivers for both variables (Figure S3).

**FIGURE 2:**
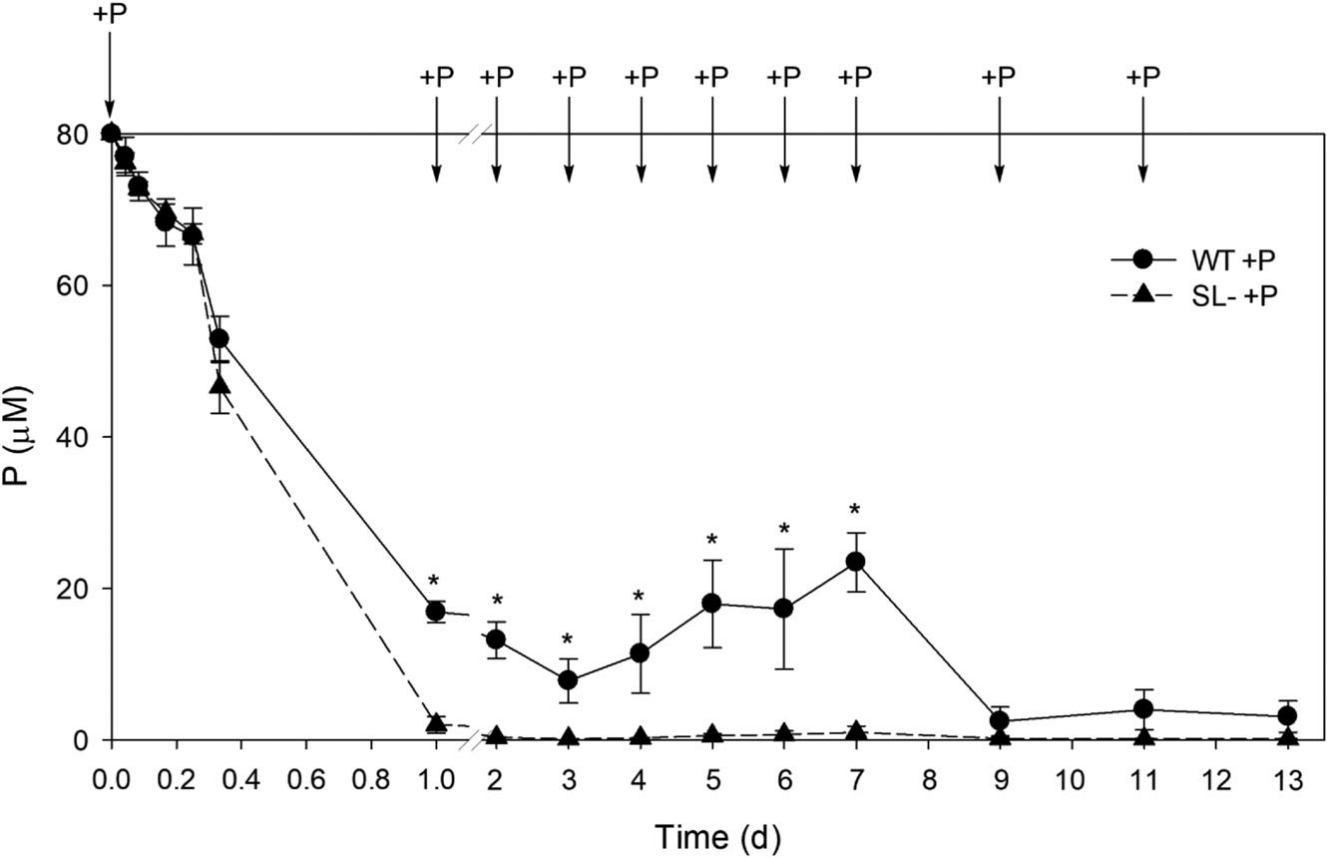
Residual P content in the exudates of wild‐type (WT) and SL‐depleted (SL–) tomato plants during the 13 days of hydroponic growth in a nutrient solution containing 80 μM Pi. Arrows indicate the substitution of the nutrient solution and restoration of the original Pi concentration (80 μM). Values are expressed as μmol of P L^−1^ of exudate solution. Each value represents the mean of four replicates (± SE). Significant differences between treatments (*p* < .05) were indicated with an asterisk

The kinetic of P depletion (Figure 2) revealed a greater consumption of P in the growth medium by SL‐depleted plants compared with the wild‐type. Such a behaviour was evident in the first 7 days, when the nutrient solution was replaced every day (at the arrows), restoring the original P concentration of 80 μM. Thereafter, when the nutrient solution was replaced every 2 days, P was almost completely removed by wild‐type plants and fully taken up by SL‐depleted plants (Figure 2).

Neither the genotype nor the P regime influenced the content of other major elements like C or N in wild‐type plants (Table 2). Conversely, more N accumulated in SL‐depleted plants under +P than –P conditions (Table 2), probably due to the higher plant DW biomass (data not shown). The C/P and N/P ratios were dramatically high for both plant genotypes in P‐depleted treatments (Table 2), reflecting the lower P content of their tissues, both above‐ and belowground.

**TABLE 2:** Carbon (C) and nitrogen (N) contents and C/P and C/N ratios of wild‐type (WT) and SL‐depleted (SL–) tomato plants after 13 days of hydroponic culture with (+P, 80 μM) or without (–P, 0 μM) Pi, followed by 24 hr in deionized water

	C content (g plant^−1^)	N content (mg plant^−1^)	C/P	N/P
WT +P	0.89 ± 0.07	92.94 ± 5.54^ab^	157 ± 8^b^	16.2 ± 0.5^b^
WT –P	0.91 ± 0.11	89.91 ± 8.71^ab^	569 ± 23^a^	56.9 ± 1.8^a^
SL– +P	1.14 ± 0.13	112.73 ± 9.76^a^	181 ± 17^b^	17.9 ± 1.2^b^
SL– –P	0.77 ± 0.08	75.53 ± 10.08^b^	568 ± 23^a^	56.6 ± 3.8^a^

*Note*: Each value represents the mean of four replicates (± SE). Different letters indicate significant differences between treatments (*p* < .05).

### 3.3 Acid phosphatase and phytase enzymatic activity in tomato roots

**FIGURE 3:**
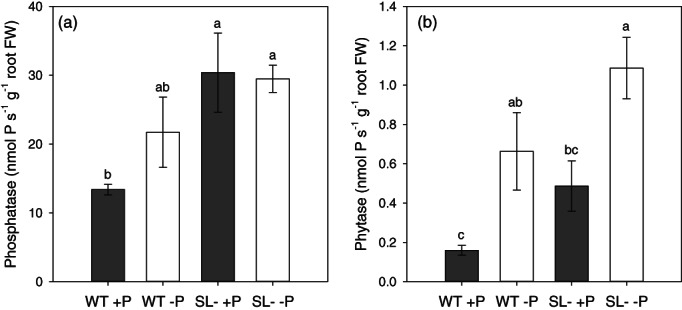
(a) Phosphatase and (b) phytase activity in roots of wild‐type (WT) and SL‐depleted (SL–) tomato plants after 13 days of hydroponic culture with (+P, 80 μM) or without (–P, 0 μM) Pi, followed by 24 hr in deionized water. Each value represents the mean of four replicates (± SE). Different letters above bars indicate significant differences between treatments (*p* < .05)

The activity of acid phosphatase was lower in wild‐type than in SL‐depleted plants, the least being in wild‐type plants receiving P and tending to increase under P‐starvation, as expected (Figure 3a). Conversely, phosphatase activity was elevated in SL‐depleted plants, either grown under +P or –P conditions, with values comparable to those of wild‐type plants under –P. In addition, phytase activity was more pronounced in P‐starved than in P‐sufficient plants (Figure 3b). Although no significant differences were evident between plant genotypes under the same P conditions, phytase activity in SL‐depleted plants showed a trend to overall higher values than in the wild‐type.

### 3.4 High‐affinity P transporters expression and regulators

**FIGURE 4:**
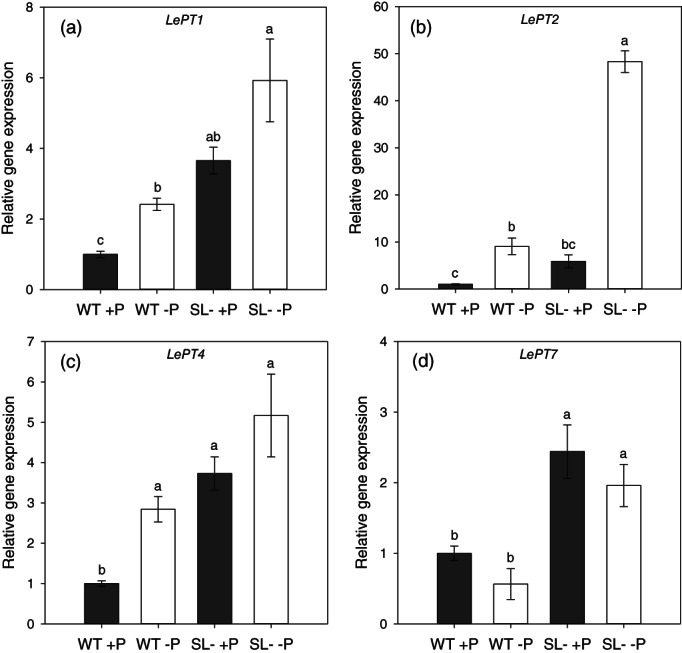
Relative gene expression of phosphate transporters (a) *LePT1*, (b) *LePT2*, (c) *LePT4* and (d) *LePT7* in roots of wild‐type (WT) and SL‐depleted (SL–) tomato plants after 13 days of hydroponic culture with (+P, 80 μM) or without (–P, 0 μM) Pi, followed by 24 hr in deionized water. Each value represents the mean of four replicates (± SE). Different letters above bars indicate significant differences between treatments (*p* < .05)

The genes encoding high‐affinity P transporters *LePT1*, *LePT2* and *LePT4* were up‐regulated under P shortage in both tomato plant genotypes, with maximum values of relative gene expression detected in SL‐depleted plants (Figure 4a–c). In contrast, transcription of the gene coding for *LePT7* was apparently not up‐regulated by P depletion in either plant genotype (Figure 4d). Interestingly the *LePT1*, *LePT2*, *LePT4* and *LePT7* transcript accumulation was significantly higher in SL‐depleted than in wild‐type plants both under +P and –P conditions (Figure 4a–d).

**FIGURE 5:**
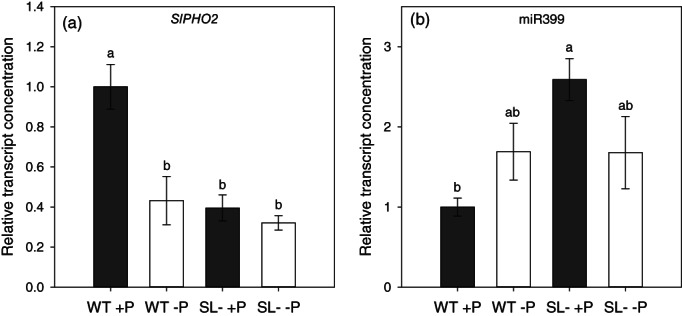
Relative (a) transcript concentration of *SlPHO2* and (b) concentration of mature miR399 in roots of wild‐type (WT) and SL‐depleted (SL–) tomato plants after 13 days of hydroponic culture with (+P, 80 μM) or without (–P, 0 μM) Pi, followed by 24 hr in deionized water. Each value represents the mean of four biological replicates (± SE). Different letters above bars indicate significant differences between treatments (*p* < .05)

The amounts of mature miR399 and *SlPHO2* transcripts diverged in the tomato root samples in this study (Figure 5). *SlPHO2* transcription was down‐regulated in roots of plants containing the highest amounts of P transporter transcripts (SL‐depleted roots, and P‐starved wild‐type roots; Figure 5a). Conversely, the concentration of mature miR399 was the lowest in P‐replete wild‐type plants, and higher in –P wild‐type and SL‐depleted plants under both P conditions (Figure 5b).

## 4 ROOT EXUDATES COMPOSITION AND KINETICS

### 4.1 Root acidification capacity and protons exudation kinetics

**FIGURE 6:**
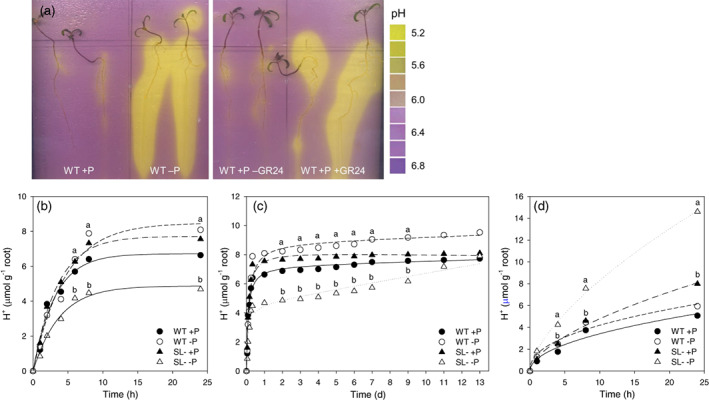
(a) Bromocresol purple assay for the acidification capacity of the roots of wild‐type (WT) tomato plants subjected to different P regimes (+P *vs* –P as positive controls, left‐hand panel) or a pre‐treatment with excess SLs under +P conditions (untreated control *vs* seedlings pre‐treated with 5 μM GR24, right‐hand panel). (b–d) Proton (H^+^) content in the exudates of wild‐type and SL‐depleted (SL–) tomato plants during (b) the first 24 hr and (c) the 13 days of hydroponic culture treatment with (+P, 80 μM) or without (–P, 0 μM) Pi. (d) Proton exudation during the 24 hr in deionized water. Values are expressed as cumulative pools and each value represents the mean of four replicates. Different letters above points indicate significant differences between treatments (*p* < .05). For the sake of graph readability, SEs are not reported

The effect of SLs on the root ability to acidify the rhizosphere was at first tested with a semi‐quantitative *in vitro* assay exploiting the pH indicator bromocresol in the agarized medium (Figure 6a). The results showed that the presence of an excess SL in the culture medium enhanced the acidification halo surrounding the roots of wild‐type tomato seedlings to an extent similar to P deprivation. Following this preliminary indication, we set out to quantify the proton cumulative release in the exudates of wild‐type and SL‐depleted plants during hydroponic growth in the short‐ (first 24 hr, Figure 6b) and long‐term (13 days, Figure 6c). Cumulative amounts of proton exudation during the 24 hr‐growth in water at the end of the hydroponic experiment were also quantified (Figure 6d). Cumulative data were fitted according to the equations reported in Table 3, together with the relative kinetic parameters.

**TABLE 3:** Parameters of the mathematical functions fitted to cumulative proton (H^+^), dissolved organic C (DOC) and organic acid anions data, as reported in Figures 6, 7, 8

H^+^							
Time	Fitted equation	Plant typeand treatment	Parameters				
			a	b	c	R^2^	p
0–24 hr	^α^ $y=a\left(1-e^{-\mathrm{bx}}\right)$	WT +P	6.71 ± 0.37	0.31 ± 0.05	‐	0.976	<.0001
		WT –P	8.47 ± 0.58	0.22 ± 0.04	‐	0.971	<.0001
		SL– +P	7.72 ± 0.25	0.29 ± 0.03	‐	0.992	<.0001
		SL– –P	4.87 ± 0.21	0.27 ± 0.03	‐	0.990	<.0001
0–13 days	^β^ $y=\frac{\mathrm{ax}}{b+x}+\mathrm{cx}$	WT + P	7.27 ± 0.28	0.09 ± 0.01	0.03 ± 0.03	0.992	<.0001
		WT –P	8.89 ± 0.44	0.13 ± 0.02	0.04 ± 0.05	0.968	<.0001
		SL– +P	8.28 ± 0.28	0.09 ± 0.01	0.02 ± 0.03	0.979	<.0001
		SL– –P	4.59 ± 0.29	0.08 ± 0.02	0.22 ± 0.04	0.964	<.0001
0–24 hr (water)	^γ^ $y=ax^{b}$	WT +P	1.10 ± 0.28	0.49 ± 0.09	‐	0.960	.0035
		WT –P	1.54 ± 0.29	0.44 ± 0.07	‐	0.971	.0021
		SL– +P	1.20 ± 0.16	0.60 ± 0.06	‐	0.993	.0002
		SL– –P	1.86 ± 0.14	0.65 ± 0.03	‐	0.998	<.0001
DOC							
0–24 hr	^δ^ $y=\frac{\mathrm{ax}}{b+x}$	WT + P	3.37 ± 0.14	5.05 ± 0.56	‐	0.993	<.0001
		WT –P	3.29 ± 0.12	4.63 ± 0.44	‐	0.995	<.0001
		SL– +P	2.13 ± 0.01	4.58 ± 0.49	‐	0.994	<.0001
		SL– –P	3.43 ± 0.11	5.04 ± 0.02	‐	0.997	<.0001
0–13 days	^β^ $y=\frac{\mathrm{ax}}{b+x}+\mathrm{cx}$	WT +P	3.15 ± 0.18	0.22 ± 0.04	0.39 ± 0.02	0.992	<.0001
		WT –P	3.00 ± 0.14	0.19 ± 0.03	0.41 ± 0.02	0.995	<.0001
		SL– +P	1.82 ± 0.07	0.16 ± 0.02	0.31 ± 0.01	0.998	<.0001
		SL– –P	3.02 ± 0.09	0.19 ± 0.02	0.44 ± 0.01	0.998	<.0001
0–24 hr (water)	^γ^ $y=ax^{b}$	WT +P	0.27 ± 0.01	0.37 ± 0.02	‐	0.998	<.0001
		WT –P	0.23 ± 0.03	0.53 ± 0.06	‐	0.985	.0008
		SL– +P	0.14 ± 0.02	0.51 ± 0.05	‐	0.990	.0004
		SL– –P	0.26 ± 0.01	0.38 ± 0.02	‐	0.998	<.0001
Organic acid anions							
*Oxalate* 0–13 days	^β^ $y=\frac{\mathrm{ax}}{b+x}+\mathrm{cx}$	WT + P	5.08 ± 0.21	0.22 ± 0.03	0.21 ± 0.02	0.997	<.0001
		WT –P	4.54 ± 0.30	0.30 ± 0.08	0.30 ± 0.03	0.997	<.0001
		SL– +P	4.41 ± 0.29	0.22 ± 0.06	0.21 ± 0.03	0.994	<.0001
		SL– –P	6.35 ± 0.30	0.21 ± 0.04	0.31 ± 0.03	0.997	<.0001
*Succinate* 0–13 days	^β^ $y=\frac{\mathrm{ax}}{b+x}+\mathrm{cx}$	WT + P	0.06 ± 0.01	1.09 ± 0.36	4.0·10^−3^ ± 7·10^−4^	0.999	.0015
		WT –P	0.09 ± 0.03	0.81 ± 0.62	3.9·10^−3^ ± 2·10^−5^	0.987	.0128
		SL– +P	0.06 ± 0.01	1.72 ± 0.35	3.3·10^−3^ ± 4·10^−5^	0.999	.0004
		SL– –P	0.11 ± 0.03	0.96 ± 0.57	2.7·10^−3^ ± 2·10^−4^	0.991	.0092

^α^ a, asymptotic H^+^ maximum; b, initial rate of H^+^ release.

^β^ a, asymptotic DOC/H^+^/organic acid anion maximum; b, initial rate of DOC/H^+^/organic acid anion release; c, net DOC/H^+^/organic acid anion increase in the linear range of the equation.

^γ^ a, initial rate of DOC/H^+^ release; b, asymptotic DOC/H^+^ value.

^δ^ a, asymptotic DOC maximum; b, initial rate of DOC release.

Proton release by plants reached a plateau within 24 hr (Figure 6b). Phosphorus‐depleted wild‐type plants, in particular, exuded slightly more protons reaching 8.47 μmol H^+^ g^−1^ of root in 24 hr (Table 3). Conversely, SL‐depleted plants were very low in proton release under –P (4.87 μmol H^+^ g^−1^ root, Table 3), but exhibited fast kinetics, similar to that of wild‐type and SL‐depleted plants under +P, as indicated by the *b* value. Intermediate plateau values, at roughly 7 μmol H^+^ g^−1^ root, were associated with P‐supplied plant genotypes. Greater proton release by –P wild‐type plants was definitely confirmed in the two‐week interval, with values slightly but constantly increasing until the end of the experiment (Figure 6c). The amount of protons liberated by SL‐depleted plants under –P was instead almost steady until 4 days, but then started increasing quickly, as indicated by the highest *c* value (Table 3). Thus, it reached a final value similar to wild‐type and SL‐depleted plants under +P, for which a plateau of proton extrusion was accomplished earlier (after 1 and 7 days, respectively) at around 8 μmol H^+^ g^−1^ root (Figure 6c). When plants were transferred to water, the proton release increased exponentially, without approaching any plateau (Figure 6d). Phosphorus‐starved SL‐depleted plants released the highest amount of protons with very fast kinetics, followed by +P plants of the same genotype (Table 3, Figure 6d). The proton release kinetics of wild‐type plants in different P treatments was similar, but the proton exudation was more intense under –P (Figure 6d, Table 3).

### 4.2 Kinetics of dissolved organic C and organic acid exudation

**FIGURE 7:**
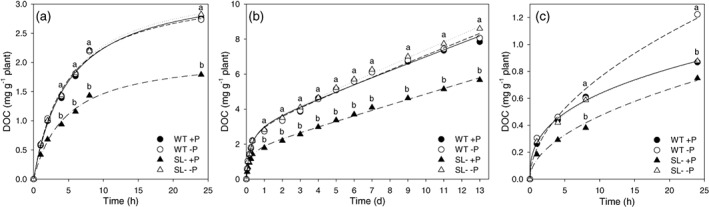
Dissolved organic C (DOC) content in the exudates of wild‐type (WT) and SL‐depleted (SL–) tomato plants during (a) the first 24 hr and (b) the 13 days of hydroponic culture treatment with (+P, 80 μM) or without (–P, 0 μM) Pi. (c) Proton exudation during the 24 hr in deionized water. Values are expressed as cumulative pools and each value represents the mean of four replicates. Different letters above points indicate significant differences between treatments (*p* < .05). For the sake of graph readability, SEs are not reported [Colour figure can be viewed at wileyonlinelibrary.com]

The kinetics of DOC exudation was evaluated at the same sampling times reported earlier for proton release (Figure 7a–c). In the first 24 hr, DOC in the solution increasingly built up until it reached a plateau (Figure 7a). Strigolactone‐depleted plants exuded less organic C when P‐replete, reaching a plateau of 2.13 mg C g^−1^ plant in 24 hr, with similar kinetics to that of P starved wild‐type plants, as indicated by the similar *b* value (Table 3). The two genotypes shared a similar pattern of C exudation under P shortage (Figure 7a), attaining a plateau at around 3.4 mg C g^−1^ plant (Table 3). However, the lower *b* values observed for –P wild‐type plants and +P SL‐depleted plants indicated faster release kinetics and faster plateauing (Table 3). In the long term (2 weeks), C exudation was comparable between SL‐depleted and wild‐type plants under –P, except for the fact that the former slightly increased C release after 9 days (Figure 7b). Because the amount of C in the solution continuously increased during 13 days, a hyperbolic equation was required to fit the data (Table 3). The net DOC increase, described by the *c* parameter, was comparable between wild‐type plants of either +P and –P treatments, but was slightly higher for SL‐depleted plants under –P (Table 3). Strigolactone‐depleted plants exhibited low organic C exudation when P was supplied, with minimum increase over time (lowest *c* value, Table 3). After being transferred to water, each genotype released more DOC when P‐depleted, with maximum values measured in the wild‐type (Figure 7c). In this case, C exudation increase was even more pronounced, and data were fitted using a power equation (Table 3). The highest amount of DOC was exuded by P‐depleted wild‐type plants (Figure 7c), while the slowest C exudation was measured in SL‐depleted plants under +P (Table 3).

**FIGURE 8:**
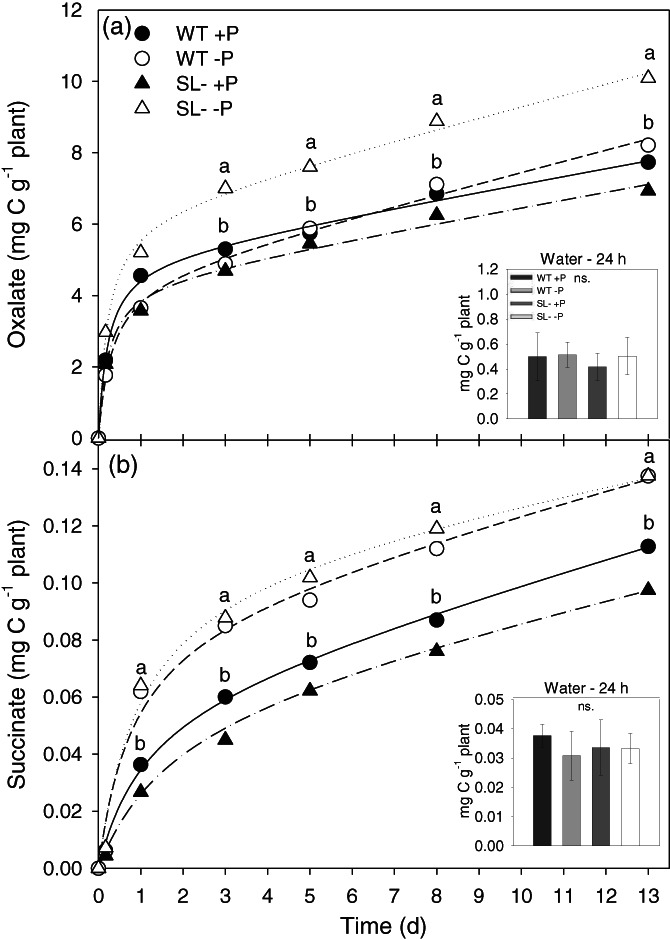
(a) Oxalate and (b) succinate contents in root exudates of wild‐type (WT) and SL‐depleted (SL–) tomato plants during the 13 days of hydroponic culture treatment with (+P, 80 μM) or without (–P, 0 μM) Pi. Values are expressed as cumulative pools of exuded C, in mg g^−1^ of plant DW. Different letters above points indicate significant differences between treatments (*p* < .05). Inset graphs: average concentration of the respective anions in the root exudates of wild‐type and SL‐depleted plants after 24 hr in deionized water. Each value represents the mean of four replicates. No statistically significant differences were observed

Qualitative and quantitative analyses of organic acid anions measured along the 13 days of P treatment and one more day in water revealed variable amounts of several types of these compounds in the root exudates of both wild‐type and SL‐depleted plants. Among detected organic acids, the most abundant were oxalate (Figure 8a) and succinate (Figure 8b). Similar to the trend reported for DOC flow from roots, cumulative exudation of oxalate and succinate anions increased over time, without reaching a plateau. In addition, the content of these major organic acids anions was lower in SL‐depleted plants under +P compared with the same –P genotype and to the wild‐type. Phosphorus‐deficient SL‐depleted plants exuded the highest amount of oxalate, with faster kinetics than the other plants (Table 3) and values significantly higher after a few hours since the beginning of the experiment (around 4 hr, Figure 8a). Conversely, wild‐type plants released comparable amounts of this compound during the whole experiment, irrespective of P supply. In addition, no significant differences in oxalate content of root exudates were evident between SL‐depleted and wild‐type plants after 24 hr of water treatment (Figure 8a, small box). The P treatments proposed in this work affected the exudation of succinate in a similar way for both tomato lines used, with P starvation inducing an increase since day 1 in both genotypes (Figure 8b, Table 3).

## 5 DISCUSSION

### 5.1 Strigolactone deficiency affects plant responses to P supply

The phenotype of the SL‐depleted plants used in this study has been described in the past, limited to morphology and physiology under P‐replete conditions (Visentin et al., 2020; Vogel et al., 2010) and to fine root morphology and tip anatomy under both P‐replete and P‐deplete conditions (Santoro et al., 2020). Our study extended their characterization under P starvation, and complemented it with data on the allocation of macroelements (C, N and P) and on the effect of exogenous SLs on PAE and PUtE. Strigolactone‐depleted plants preferentially allocated P in the aerial part, possibly to sustain their more branched phenotype. Therefore, the lack of these hormones apparently did not interfere with the process of P remobilization between organs in the plant. Delayed leaf senescence symptoms in SL‐depleted plants irrespective of P availability confirmed a role for SLs as positive regulators of leaf senescence, being able to activate the ethylene‐mediated signalling pathway reallocating nutrients from old to young, developing tissues (Czarnecki et al., 2013; Ueda & Kusaba, 2015; Yamada et al., 2014; Yang et al., 2019).

The higher P content of SL‐depleted shoots under +P compared with the wild‐type was justified by their elevated PAE, which determined faster and complete consumption of P from the nutrient solution. Consistently and in a complementary way, excess SLs delivered as GR24 increased plant PUtE and decreased PAE. In contrast, wild‐type plants consumed less P, about 80% of the amount provided during the first day, and between 70% and 90% of P on average when the nutrient solution was renewed every day. The higher, time‐dependent demand of P by SL‐depleted plants may be due to altered perception of exogenous P and/or to disturbance of the systemic PSR control, consistent with the altered responses to this nutrient and the changes in root system architecture and tip anatomy reported by Santoro et al. (2020). Coherently with better P removal from the nutrient solution, gene transcripts of the root high‐affinity P transporters *LePT1*, *LePT2*, *LePT4* and *LePT7* were more concentrated under +P in SL‐depleted plants than in the wild‐type. *LePT1*, *LePT2* and *LePT4* were induced by P starvation in wild‐type plants, but not in SL‐depleted plants, while *LePT7* behaved differently, as it was not up‐regulated by P starvation in either plant genotype. In particular, among the eight PHT‐encoding genes identified in *S. lycopersicum*, *LePT1* and *LePT7* are ubiquitously expressed in plant tissues, especially in roots and leaves and under low P conditions, while *LePT4* is usually sharply up‐regulated in the roots colonized by AMF under low P (Chen et al., 2014). Our results are partly in agreement with the findings of Gamir et al. (2020), as far as inducibility of *LePT2* by P starvation in the wild‐type. However, they are apparently in contrast with their observation that the transcriptional induction of *LePT2* and of the PSR regulator miR399, and the down‐regulation of *SlPHO2*, were compromised in the tomato SL‐depleted line *SlCCD8*‐RNAi L04 when compared with the wild‐type under low P. However, the very different conditions under which these results were obtained (80 *vs* 800 μM Pi for P‐sufficient condition, and 0 *vs* 200 μM Pi for the P‐depleted condition in our work and in Gamir et al., 2020, respectively) highlight how the regulation of P transporters in SL‐depleted plants is strongly dependent on the P concentration applied and is modified when the growth conditions resemble the more realistic, low concentrations of available P in soils. Such differences in P supply may have triggered diverse regulatory signals of PHT transporters in the two genotypes in our study. It is conceivable that the P concentrations we applied were sufficiently low to be perceived as an almost complete absence of P by the SL‐depleted plants, resulting in the activation of the core PSR module (higher miR399 and PHT transcripts, lower *SlPHO2*) at P levels that are still perceived as sufficient by wild‐type plants.

The hypothesized higher susceptibility to P shortage displayed by SL‐depleted plants and consequent altered regulatory mechanisms is further supported by the higher phytase and acid phosphatase activities measured in their roots. Increased phosphatase activity might be indicative of more intense P release from P‐containing cellular constituents, such as membrane phospholipids, to promote P recycling in the plant (Bozzo, Dunn, & Plaxton, 2005; Vance et al., 2003). Elevated activity of phytases instead suggests an attempt of plants to mine P from phytate, which is a major organic P form in soils (Celi & Barberis, 2004), or to use the internal phytate‐derived P, given that this compound is the principal storage form of P in many plant tissues (Baldwin, Athikkattuvalasu, & Raghothama, 2001; Brinch‐Pedersen, Sorenson, & Holm, 2002; Hayes, Richardson, & Simpson, 2000). A strong positive correlation between P and N contents was found in our plants (Pearson correlation coefficient ρ = 0.619, *p* < .01) in accordance with previous studies (de Groot, Marcelis, van den Boogaard, Kaiser, & Lambers, 2003). The activity of P‐mobilizing enzymes in SL‐depleted plants was higher than in the P‐supplied wild‐type, but overall comparable to that of P‐depleted wild‐type plants. Such a result indicates that while the typical PSRs are activated in SL‐depleted plants under +P perhaps because of altered P signalling and perception, the onset of these responses in the same genotype when P‐starved was not as intense as conceivable from its elevated P requirement for supporting optimal growth. The R/S ratio and PUtE of –P SL‐depleted plants were indeed comparable to those of –P wild‐type plants, and higher than +P plants. The modification of resource distribution in favour of belowground biomass production over shoot growth negatively correlated with the plant P content (ρ = 0.867, *p* < .01) in both genotypes. This is a typical response to P starvation intended to increase soil exploration and P uptake by roots (Aziz et al., 2014; Niu et al., 2013; Ramaekers, Remans, Rao, Blair, & Vanderleyden, 2010); the fact that it was observed in both wild‐type and SL‐depleted plants confirms that the differences between these genotypes observed by Santoro et al. (2020) under –P conditions were likely due to differences in root anatomy and topology rather than biomass allocation.

### 5.2 Strigolactones modify exudate composition under P depletion

The results discussed so far suggest that SL‐depleted plants activate some of the strategies that are usually adopted to respond to P shortage when the concentration of P in the nutrient solution resembles what is found in natural soils, and when wild‐type plants still are in P‐replete acquisition mode. In light of these results, we sought to understand whether this increased P demand by SL‐depleted plants was also translated in a different release of protons and organic acid anions.

We observed a fast proton release in the first 24 hr, followed by slower kinetics in the long term, maintaining, however, significant differences in kinetic parameters and intensity of exudation between genotypes and P‐supply regimes. Wild‐type plants responded indeed more promptly than SL‐depleted plants to P‐starvation, with a higher proton release. These results are nicely complemented by the observation that excess SLs in the form of GR24 can trigger rhizosphere acidification also in wild‐type plants under P‐replete conditions, supporting the notion that SLs promote proton excretion in the exudates. Already after 24 hr, wild‐type plants under –P released the highest amount of protons, almost double than SL‐depleted plants, and maintained this high exudation trend in the long term. Conversely, the initially lower exudation by SL‐depleted plants under –P was followed by an increase after 4 days, reaching values comparable to the plateau attained by +P plants. This could be due to a delayed activation of factors acting in parallel with SLs in the signalling pathway activated under –P. In fact, although the loss of sensitivity of plant response to low P by SL mutants was reported in rice (Sun et al., 2014) and *Arabidopsis* (Mayzlish‐Gati et al., 2012), at least in terms of root architecture modifications, it was also shown that the SL mutants *more axillary growth* (*max*) *2–1* and *max4‐1* were able to increase root hair production in response to low P over time, eventually reaching wild‐type levels (Mayzlish‐Gati et al., 2012). In addition, the proton extrusion from P‐starved SL‐depleted plants strongly intensified when they were transferred to water. We hypothesize that they may have suffered more than wild‐type plants when all other nutrients were no longer available, and as a consequence, they boosted proton release in the attempt to forage for more nutrients.

Conversely to proton exudation, DOC exudation continuously increased even in the long term; weaker differences were observed between +P and –P wild‐type plants, in line with other studies carried out on tomato (Neumann & Römheld, 1999). It is also possible that the P dosages applied did not allow discriminating differences in organic acid exudation, or that plants rather co‐activated alternative P‐acquiring strategies to prevent excessive C losses. The low DOC exudation observed in SL‐depleted plants under +P might be in part due to a feedback repression exerted by higher P accumulation in the shoot. When plants were transferred to water to stimulate further exudation, DOC release kept increasing over time, and wild‐type plants that were coming from –P conditions released more exudates, while SL‐depleted plants coming from +P had the lowest exudation rates. The increased C exudation by P‐depleted, wild‐type plants could be the result of a forced release of re‐accumulated organic acids, as proposed by Tiziani, Pii, Celletti, Cesco, and Mimmo (2020) using ^13^C‐labelled molecules. Organic acid anions analysis revealed that the majority of C consisted in oxalic acid anions, whose exudation kinetics clearly matched that of DOC. Oxalic acid is the simplest dicarboxylic acid, with high acidity (pK_a1_ = 1.23) and strong chelating capacity for Ca^2+^, Al^3+^ and Fe^3+^ (Ryan, Delhaize, & Jones, 2001). Because of its chemical features, its release by tomato roots may be energetically favoured over other metabolic intermediates, such as citrate and malate (Zhao & Wu, 2014). This was particularly clear in SL‐depleted plants under –P conditions. Conversely, succinate was exuded based on P availability rather than genotypic differences.

In conclusion, our results highlight that SL‐depleted plants did not efficiently activate most mechanisms associated with PSRs under P shortage, except for the up‐regulation of P transporters and increased activity of P‐solubilizing enzymes, which was over‐activated under P‐replete conditions already. The reduced SL biosynthesis had negative effects under either P conditions. When P was supplied, SL‐depleted plants activated molecular, physiological and biochemical responses to sustain elevated P uptake, with great C and N costs and energy dissipation for building up P transporters and P‐mobilizing enzymes, which in turn may have been responsible for lower C and organic acid exudation. Under –P conditions, the same genotype was less efficient at responding to P shortage than the wild‐type, with unaltered expression of some P transporters and enzyme activity. This reduced response was also reflected in the delayed activation of root proton exudation, compared with the faster and higher release by P‐starved, wild‐type plants.

We conclude that SLs are master kinetic regulators of PSR in tomato, and their silencing might be responsible for both suboptimal nutritional outcomes under P depletion, and unbalanced control of P uptake when P is available.

## CONFLICT OF INTEREST

The authors declare no conflict of interest. The funders had no role in the design of the study; in the collection, analyses or interpretation of data; in the writing of the manuscript, or in the decision to publish the results.

## Supporting information


**Figure S1.** Wild‐type (WT) and SL‐depleted (SL–) tomato plants developmental response to P treatments. Plants were grown in quartz sand for 45 days with a nutrient solution containing 80 μM Pi, then transferred to hydroponic culture with (+P, 80 μM) or without (–P, 0 μM) Pi for 13 days, followed by 24 hr in deionized water.
**Figure S2.** P translocation (relative P shoot/P root) from roots to shoots of wild‐type (WT) and SL‐depleted (SL–) tomato plants after 13 days of hydroponic culture with (+P, 80 μM) or without (–P, 0 μM) Pi, followed by 24 hr in deionized water. Each value represents the mean of four replicates (± SE). Different letters above bars indicate significant differences between treatments (*p* < 0.05).
**Figure S3.** (a, b) PAE and (c, d) PUtE values in roots (a, c) and shoots (b, d) of potted plants grown in the greenhouse at 125 μM Pi and treated, or not, with 5 μM GR24. Each value represents the mean of four replicates (± SE). Different letters above bars indicate significant differences between treatments (*p* < .05). PAE and PUtE are reported as relative values according to the formulas indicated in the main text.
**Table S1.** List of primers used in this work.

## Data Availability

The data that support the findings of this study are available from the corresponding author upon reasonable request.
