# Supplementary material for: Strigolactones affect phosphorus acquisition strategies in tomato plants
Source: Plant Cell Environ. 2021 Aug 25;44(11):3628–42. doi: 10.1111/pce.14169 (PMC9290678; doi:10.1111/pce.14169)
Supplement: Supplementary file 1 — Figure S1. Wild‐type (WT) and SL‐depleted (SL–) tomato plants developmental response to P treatments. Plants were grown in quartz sand for 45 days with a nutrient solution containing 80 μM Pi, then transferred to hydroponic culture with (+P, 80 μM) or without (–P, 0 μM) Pi for 13 days, followed by 24 hr in deionized water. Figure S2. P translocation (relative P shoot/P root) from roots to shoots of wild‐type (WT) and SL‐depleted (SL–) tomato plants after 13 days of hydroponic culture with (+P, 80 μM) or without (–P, 0 μM) Pi, followed by 24 hr in deionized water. Each value represents the mean of four replicates (± SE). Different letters above bars indicate significant differences between treatments (p < 0.05). Figure S3. (a, b) PAE and (c, d) PUtE values in roots (a, c) and shoots (b, d) of potted plants grown in the greenhouse at 125 μM Pi and treated, or not, with 5 μM GR24. Each value represents the mean of four replicates (± SE). Different letters above bars indicate significant differences between treatments (p < .05). PAE and PUtE are reported as relative values according to the formulas indicated in the main text. Table S1. List of primers used in this work. [file PCE-44-3628-s001.docx]

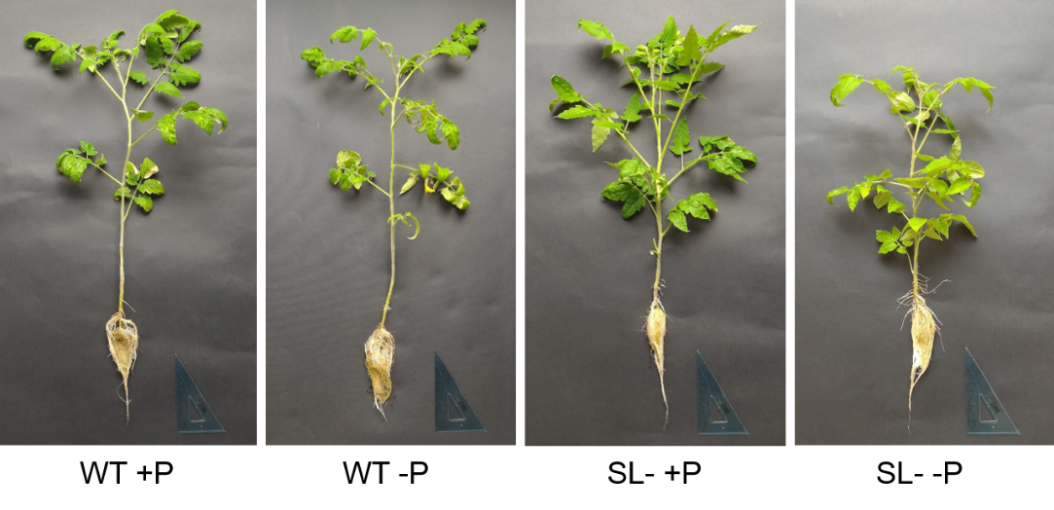


**Figure S1:** Wild-type (WT) and SL-depleted (SL–) tomato plants developmental response to P treatments. Plants were grown in quartz sand for 45 days with a nutrient solution containing 80 µM Pi, then transferred to hydroponic culture with (+P, 80 µM) or without (–P, 0 µM) Pi for 13 days, followed by 24 h in deionized water.

**
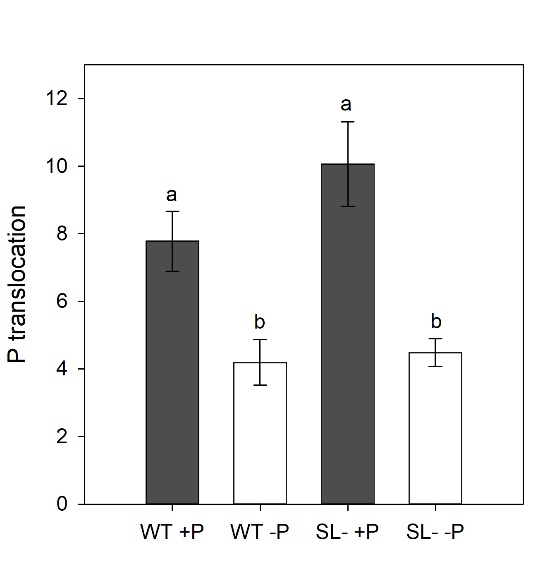
**

**Figure S2:** P translocation (relative P shoot/P root) from roots to shoots of wild-type (WT) and SL-depleted (SL–) tomato plants after 13 days of hydroponic culture with (+P, 80 µM) or without (–P, 0 µM) Pi, followed by 24 h in deionized water. Each value represents the mean of four replicates (± SE). Different letters above bars indicate significant differences between treatments (*p* < 0.05).


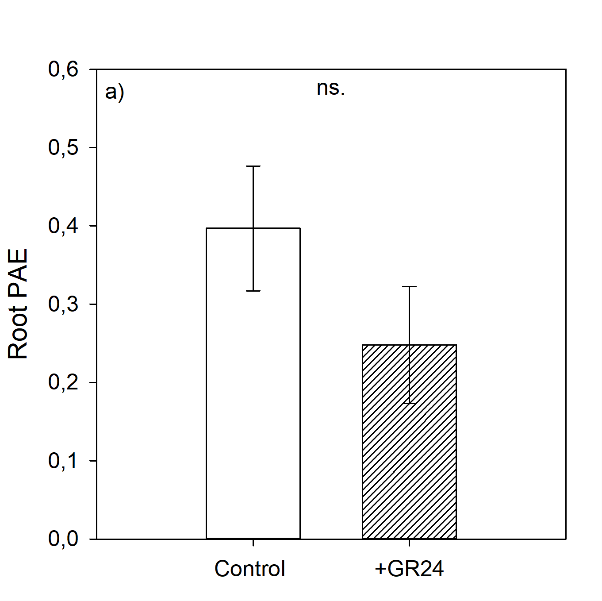

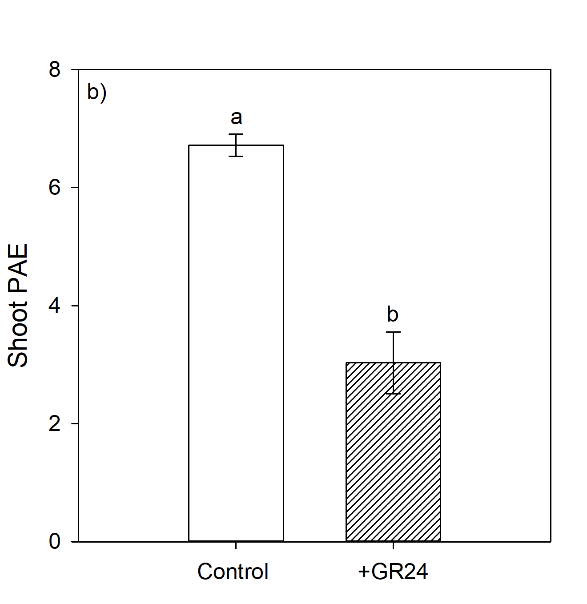

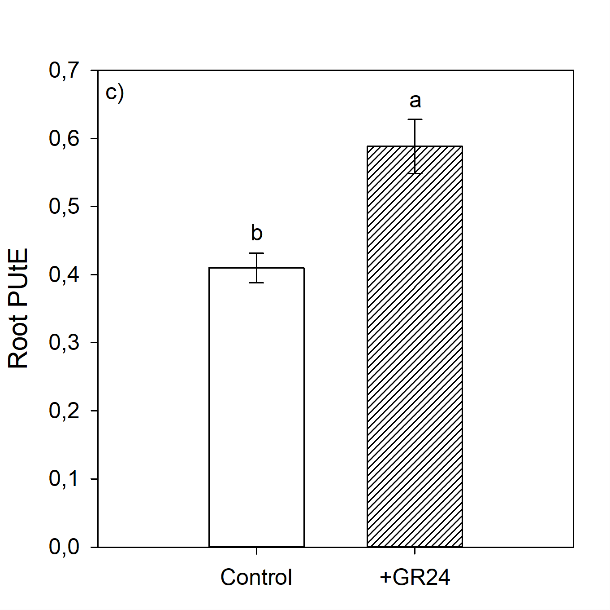

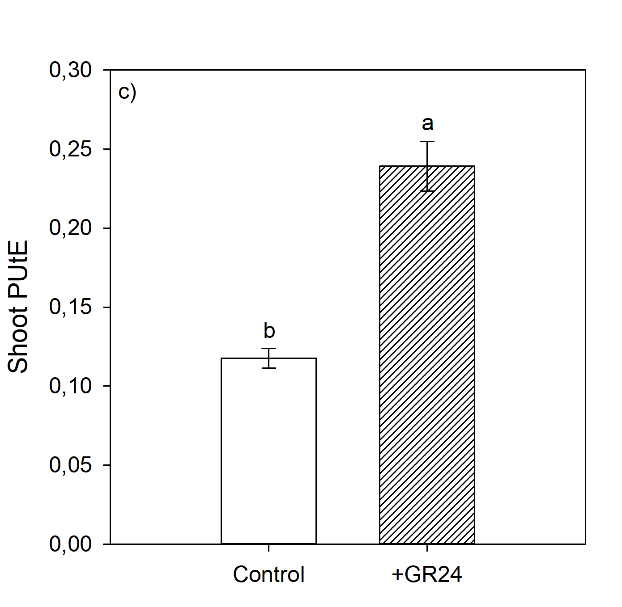


**Figure S3:** a,b) PAE and c,d) PUtE values in roots (a,c) and shoots (b,d) of potted plants grown in the greenhouse at 125 µM Pi and treated, or not, with 5 µM GR24. Each value represents the mean of four replicates (± SE). Different letters above bars indicate significant differences between treatments (*p* < 0.05). PAE and PUtE are reported as relative values according to the formulas indicated in the main text.

**Table S1**: List of primers used in this work.

| **Primer/target name** | **Sequence** | **Reference** |
| --- | --- | --- |
| stem-loop miR399 | 5'-GTCGTATCCAGTGCAGGGTCCGAGGTATTCGCACTGGATACGACTAGGGC-3 | This work |
| Mature miR399 | 5'-AACCGTTGCCAAAGGAGAG-3 | This work |
|  | 5'-GTGCAGGGTCCGAGGT-3' |  |
| *SlsnRU6* | 5'-GGGAACGATACAGAGAAGATTAGC-3’ | Visentin et al. (2020) |
|  | 5'-ACCATTTCTCGATTTGTGCGT-3’ |  |
| *SlEF-1*α | 5'-CTCCATTGGGTCG TTTTGCT-3’ | Digilio *et al.* (2010) |
|  | 5'-GGTCACCTTGGC ACCAGTTG-3’ |  |
| *SlPHO2* | 5'-AGGGTGCAAGTTCAGTCTGT-3' | This work |
|  | 5'-TCCAGCCTCCACAGCATAAA-3' |  |
| *LePT1* | 5’-TTCCTCGGTATGCTGTTCAC-3’ | This work |
|  | 5’-TTCCTCTTCCCCTTCGTTTTC-3’ |  |
| *LePT2* | 5'-AGAGGAAGCATCACAAGAAACT-3' | This work |
|  | 5'-ACCATTTTACAATAACACACTTGGC-3’ |  |
| *LePT4* | 5’-AGGGCTGGTTTCGATGATCA-3’ | This work |
|  | 5’-GGGCTCCGTGGACAAAATTT-3’ |  |
| *LePT7* | 5’-AGATTACGGTCCACTTGCCA-3’ | This work |
|  | 5’-GCCTTGGTCTTGTCTTGTGG-3’ |  |

References not included in the main list: Digilio, M.C., Corrado, G., Sasso, R., Coppola, V., Iodice, L., Pasquariello, M., Bossi, S., Maffei, M.E., Coppola, M., Pennacchio, F., Rao, R., Guerrieri, E. (2010). Molecular and chemical mechanisms involved in aphid resistance in cultivated tomato. *New Phytologist,* *187* (4), 1089-1101.
